# Supplementary material for: Interplay between Josephson and Aharonov-Bohm effects in Andreev interferometers
Source: Sci Rep. 2019 Feb 4;9:1301. doi: 10.1038/s41598-018-37653-w (PMC6361916; doi:10.1038/s41598-018-37653-w)
Supplement: Supplementary file 1 — Interplay between Josephson and Aharonov-Bohm effects in Andreev interferometers [file 41598_2018_37653_MOESM1_ESM.pdf]

# Interplay between Josephson and Aharonov-Bohm effects in Andreev interferometers

**Pavel E. Dolgirev[1,*], Mikhail S. Kalenkov[2,3], and Andrei D. Zaikin[4,5]**

[1]Skolkovo Institute of Science and Technology, Skolkovo Innovation Center, 3 Nobel St., 143026 Moscow, Russia
[2]I.E. Tamm Department of Theoretical Physics, P.N. Lebedev Physical Institute, 119991 Moscow, Russia
[3]Moscow Institute of Physics and Technology, Dolgoprudny, 141700 Moscow region, Russia
[4]Institut für Nanotechnologie, Karlsruher Institut für Technologie (KIT), 76021 Karlsruhe, Germany
[5]National Research University Higher School of Economics, 101000 Moscow, Russia
[*]pavel.dolgirev@skolkovotech.ru

Consider first an SNS junction with a normal-metal wire of length $L$ connecting two superconducting terminals. At sufficiently high energies $\varepsilon \gg \mathscr{E}_{\rm Th}$ the solution of the Usadel equation in the normal wire can be expressed as a superposition of the two independent anomalous propagators[22]:

$$\mathscr{F}_{12}(x) = \mathscr{F}_{\rm SN}\left(\frac{L}{2}+x\right)e^{i\frac{\phi}{2}} + \mathscr{F}_{\rm SN}\left(\frac{L}{2}-x\right)e^{-i\frac{\phi}{2}}, \tag{S1}$$

$$\mathscr{F}_{21}(x) = -\mathscr{F}_{\rm SN}\left(\frac{L}{2}+x\right)e^{-i\frac{\phi}{2}} - \mathscr{F}_{\rm SN}\left(\frac{L}{2}-x\right)e^{i\frac{\phi}{2}} \tag{S2}$$

where

$$\mathscr{F}_{\rm SN}(x) = -\frac{4q(1+q^2)}{(1-q^2)^2},\; q(x) = \frac{i}{1+\sqrt{2}}e^{x\sqrt{\frac{-2i\varepsilon}{D}}} \tag{S3}$$

and $x$ is the coordinate along the wire ($-L/2 \le x \le L/2$). Combining the above expressions with that for the spectral supercurrent

$$j_s = \frac{1}{4}\,{\rm Tr}\,\hat{\tau}_3\left(\hat{G}^R\partial_x\hat{G}^R - \hat{G}^A\partial_x\hat{G}^A\right) = \frac{1}{4}[\mathscr{F}_{12}\partial_x\mathscr{F}_{21} - \mathscr{F}_{21}\partial_x\mathscr{F}_{12} + c.c.] \tag{S4}$$

we readily find

$$j_s = \frac{16}{3+2\sqrt{2}}\sin\phi\left[i\sqrt{\frac{-2i\varepsilon}{D}}e^{\sqrt{\frac{-2i\varepsilon}{D}}} + c.c.\right]. \tag{S5}$$

Having established this relation, we can now turn to the symmetric cross-like geometry[11–13] with two superconducting and two normal terminals biased by the voltage $V$, cf. also Fig. 1 of the main text with $l_c = 0$ and disconnected terminal $N_3$. Integrating Eq. (S5) over energies and bearing in mind that at $T \to 0$ only states with energies $|\varepsilon| \ge eV/2$ contribute to this integral, in the limit $eV \gg \mathscr{E}_{\rm Th}$ we obtain

$$I_C^{SNS}(V) \simeq \frac{32(1+v^{-1})}{3+2\sqrt{2}}\frac{V}{R_L}e^{-v}\sin(v+v^{-1}), \tag{S6}$$

where the parameter $v$ was defined above in Eq. (17).

Note that this simple analysis totally neglects partial suppression of superconducting correlations inside the normal wires due to the presence of two extra normal terminals. The magnitude of this effect depends on the system topology and can be accounted for in a straightforward manner by formulating the matching conditions at the wire branching points. In a general form such conditions have already been discussed, e.g., in Refs.[16,19]. In essence, they are equivalent to the current conservation law interpreted as a "Kirchoff law for the Green functions" – see, e.g., Eq. (29) in Ref.[19].

Here we derive a simplified version of these matching conditions sufficient for our present purposes. To do so, we note that for energies $|\varepsilon| \gg \mathscr{E}_{\rm Th}$ and at distances from a superconductor exceeding the length scale $\sim \sqrt{D/|\varepsilon|}$ the Usadel equation can be linearized, thereby reducing to a simple wave-like equation:

$$Dd^2\mathscr{F}/dx^2 + 2i\varepsilon\mathscr{F} = 0. \tag{S7}$$

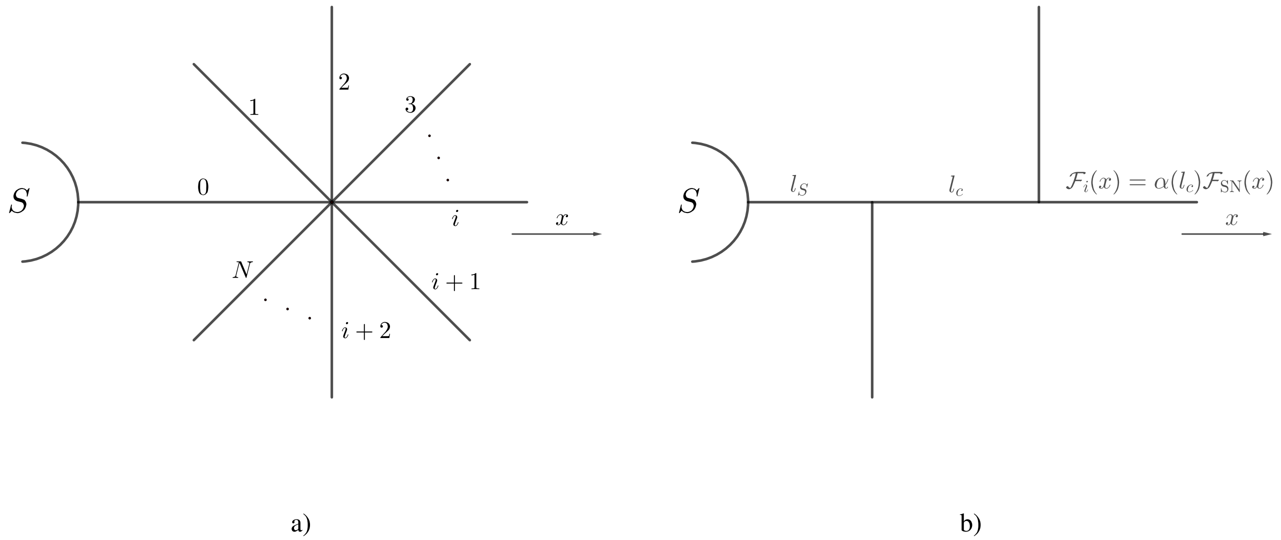


**Figure S1.** (a) A configuration with a superconducting terminal and attached normal wire(s) containing a branching point. (b) The same with varying length $l_c$ between two branching points.

As this equation is linear, the effect from the superconducting terminals can be treated independently, cf., e.g., Eqs. (S1)–(S2).

To this end let us consider a setup schematically depicted in Fig. S1a. The setup consists of one superconducting terminal attached to a normal wire which, in turn, is connected to $N$ other normal wires at some branching point located sufficiently far from the superconductor. By solving the wave-like equation (S7), we obtain the following matching conditions

$$\mathscr{F}_i(x) = \alpha \mathscr{F}_{\rm SN}(x),\ \alpha = 2\mathscr{A}_0 / \Big( \sum_{j=0}^{N} \mathscr{A}_j \Big),\ i = 1, \dots, N, \tag{S8}$$

where $\mathscr{A}_i$ denotes the cross section of the $i$-th normal wire and $x$ is a coordinate along the corresponding wire. This condition implies that the anomalous propagator $\mathscr{F}_i(x)$ inside the $i$-th wire is suppressed by the factor $\alpha$ as compared to the propagator $\mathscr{F}_{\rm SN}(x)$ in the absence of the branching point ($N = 1$).

Applying the matching condition (S8) to the setup of Fig. 1 and bearing in mind that all wire cross sections are assumed to be equal $\mathscr{A}_i = \mathscr{A}$, for the anomalous propagator $\mathscr{F}_{12}(x)$ inside the wire $l_c$ we obtain

$$\mathscr{F}_{12}(x) = \alpha \mathscr{F}_{\rm SN}\Big(\frac{L}{2} + x\Big) e^{i\frac{\phi}{2}} + \tilde{\alpha} \mathscr{F}_{\rm SN}\Big(\frac{L}{2} - x\Big) e^{-i\frac{\phi}{2}}, \tag{S9}$$

with $\alpha = \tilde{\alpha} = 2/3$ in the absence of the terminal $N_3$ (four-terminal configuration) and $\alpha = 2/3$, $\tilde{\alpha} = 4/9$ (or vice versa depending on whether $x$ is located to the left or to the right with respect to the wire $l_{N,3}$) for the five-terminal setup in Fig. 1. Accordingly, in these two cases the currents $I_C^{(4)}(V)$ and $I_{C,0}^{(5)}(V)$ get reduced respectively by the factors $4/9$ and $8/27$ compared to $I_C^{SNS}(V)$ in Eq. (S6).

Note that, strictly speaking, the latter results for the prefactors $\alpha$ and $\tilde{\alpha}$ apply only provided different branching points in the setup of Fig. 1 are located sufficiently far from each other. In order to address a more general situation let us consider the configuration depicted in Fig. S1b with the wire length $l_c$ varying from zero to large values. According to Eq. (S8), for $l_c \to 0$ we have $\alpha(0) = 1/2$ (we again assume that all wires have the same cross section), while in the limit of large $l_c$ one finds $\alpha = (2/3)^2 = 4/9$. Making use of the linearity of Eq. (S7) and the conservation of the spectral currents, we recover the complete dependence $\alpha(l_c)$:

$$\alpha(l_c) \approx 4 \left( 9 - \exp\left( 2 l_c \sqrt{\frac{-2i\varepsilon}{D}} \right) \right)^{-1}. \tag{S10}$$

This result demonstrates that in a general case the function $\alpha(l_c)$ depends on energy.

Bearing in mind the above matching conditions, for the setup in Fig. 1 we obtain

$$I_C^{(4)}(V) \simeq I_C^{SNS}(V)\begin{cases}\frac{4}{9} & \text{if } l_c^2 \gg D/(eV)\\ \frac{1}{2} & \text{if } l_c^2 \ll D/(eV)\end{cases}, \tag{S11}$$

$$I_{C,0}^{(5)}(V) \simeq I_C^{SNS}(V)\begin{cases}\frac{8}{27} & \text{if } l_c^2 \gg D/(eV)\\ \frac{2}{5} & \text{if } l_c^2 \ll D/(eV)\end{cases}. \tag{S12}$$

## References


1. W. Belzig, F.K. Wilhelm, C. Bruder, G. Schön and A.D. Zaikin, Superlatt. Microstruct. **25**, 1251 (1999).
2. A.G. Semenov, A.D. Zaikin, and L.S. Kuzmin, Phys. Rev. B **86**, 144529 (2012).
3. A.G. Semenov and A.D. Zaikin, Phys. Rev. B **91**, 024505 (2015).
4. G. Falci, D. Feinberg, and F.W.J. Hekking, Europhys. Lett. **54**, 255 (2001).
5. D. Beckmann, H.B. Weber, and H. v. Löhneysen, Phys. Rev. Lett. **93**, 197003 (2004).
6. S. Russo, M. Kroug, T.M. Klapwijk, and A.F. Morpurgo, Phys. Rev. Lett. **95**, 027002 (2005).
7. P. Cadden-Zimansky and V. Chandrasekhar, Phys. Rev. Lett. **97**, 237003 (2006).
8. M.S. Kalenkov and A.D. Zaikin, Phys. Rev. B **76**, 224506 (2007).
9. D.S. Golubev, M.S. Kalenkov, and A.D. Zaikin, Phys. Rev. Lett. **103**, 067006 (2009).
10. A.F. Volkov, Phys. Rev. Lett. **74**, 4730 (1995).
11. F.K. Wilhelm, G. Schön, and A.D. Zaikin, Phys. Rev. Lett. **81**, 1682 (1998).
12. S. Yip, Phys. Rev. B **58**, 5803 (1998).
13. J.J.A. Baselmans, A.F. Morpurgo, B.J. van Wees and T. M. Klapwijk, Nature **397**, 43 (1999).
14. H. Nakano and H. Takayanagi, Solid State Commun. **80**, 997 (1991).
15. V.T. Petrashov, V.N. Antonov, P. Delsing, and T. Claeson, Phys. Rev. Lett. **70**, 347 (1993); **74**, 5268 (1995).
16. A.V. Zaitsev, Physica B **203**, 274 (1994).
17. T.H. Stoof and Yu.V. Nazarov, Phys. Rev. B **54**, R772 (1996).
18. H. Courtois, P. Gandit, D. Mailly, and B. Pannetier, Phys. Rev. Lett. **76**, 130 (1996).
19. A.A. Golubov, F.K. Wilhelm, and A.D. Zaikin, Phys. Rev. B **55**, 1123 (1997).
20. P.E. Dolgirev, M.S. Kalenkov and A.D. Zaikin, Phys. Rev. B **97**, 054521 (2018).
21. P.E. Dolgirev, M.S. Kalenkov and A.D. Zaikin, arXiv:1806.06882.
22. A.D. Zaikin and G.F. Zharkov, Fiz. Nizk. Temp. **7**, 375 (1981) [Sov. J. Low Temp. Phys. **7**, 181 (1981)].
23. P. Dubos, H. Courtois, B. Pannetier, F.K. Wilhelm, A.D. Zaikin, and G. Schön, Phys. Rev. B **63**, 064502 (2001).
24. P. Virtanen and T.T. Heikkilä, Phys. Rev. Lett. **92**, 177004 (2004); J. Low Temp. Phys. **136**, 401 (2004).
25. M.S. Kalenkov and A.D. Zaikin, Phys. Rev. B **95**, 024518 (2017).
